# Paraneoplastic oesophageal dysmotility-renal cell carcinoma presenting as dysphagia: a case report

**DOI:** 10.4076/1757-1626-2-8170

**Published:** 2009-07-31

**Authors:** Mahesh D Bhalme, Scott E Levison, Gurvinder S Banait

**Affiliations:** 1 Department of Gastroenterology, Rochdale InfirmaryWhitehall Street, Rochdale, Lancashire, OL12 0NBUK; 2 University of ManchesterOxford Road, Manchester, M13 9PLUK; 3 Department of Gastroenterology, Royal Blackburn HospitalBlackburn, Lancashire, BB2 3HHUK

## Abstract

**Introduction:**

Dysphagia and weight loss are alarming symptoms that warrant urgent assessment.

**Case presentation:**

We present a case report of dysphagia secondary to oesophageal dysmotility attributed to a paraneoplastic manifestation of an occult renal cell carcinoma.

**Conclusion:**

We believe this patient's dysphagia was a paraneoplastic manifestation of the renal cell tumour, an association that has never been previously reported. This case demonstrates the need to look for alternative causes for dysphagia if initial investigation and treatment are unhelpful. Importantly, this must include the consideration of a paraneoplastic process secondary to an occult neoplasm.

## Introduction

Dysphagia and weight loss are alarming symptoms that warrant urgent assessment to determine its cause and initiate appropriate treatment.

Here we report a case of dysphagia secondary to oesophageal dysmotility which was attributed to a paraneoplastic manifestation of an occult renal cell carcinoma.

## Case presentation

A previously well 55 year old Caucasian male presented to gastroenterology out patients clinic in November 2003, with a three week history of worsening dysphagia to solids and weight loss of 4 kilograms. He described a sensation of food sticking at the level of the lower sternum. He had suffered in the past with mild heartburn for which he took a proton pump inhibitor on an as required basis. He was a married and worked as an accountant. He had never smoked. Physical examination including the body mass index and blood pressure was unremarkable.

Initial investigations revealed proteinuria (++) on urine dipstick, a haemoglobin of 11.8 (normal indices) with an otherwise normal blood count and film. Urea and electrolytes, estimated glomerular filtration rate, liver enzymes, thyroid stimulating hormone, random blood glucose, bone profile, haematinics and urine culture & sensitivity were normal. The ESR was 96 mm/hour. Unfortunately 24 hour urinary protein quantification was never been considered as the patient's presentation did not suggest nephrotic or nephritic syndrome.

Oesophagogastroduodenoscopy including biopsies from the mid and lower third of oesophagus were normal thus excluding eosinophilic oesophagitis.

**Figure 1.**
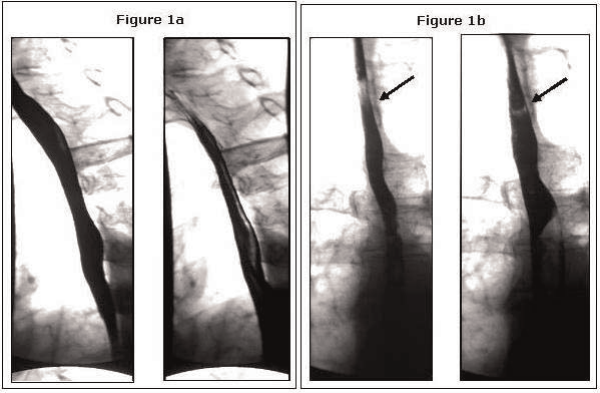
(**A**) Barium Swallow Radiograph - Liquid medium flowing freely. **(B)** Barium Swallow Radiograph - Solid medium (bread soaked in barium) is been held up in lower oesophagus.

Although a liquid barium swallow showed no abnormality, a swallow with a solid medium (bread soaked in barium) detected a significantly abnormal non-specific dysmotility of the lower oesophagus (Figure 1a,b). This was performed because the use a solid medium during a barium swallow increases the sensitivity of the test by generating a greater amplitude and duration of peristalsis [1]. The dysmotility that was seen was confirmed with oesophageal manometry.

**Figure 2.**
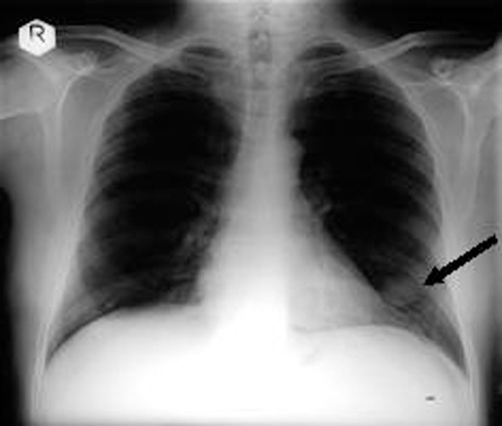
Chest radiograph showing a ‘coin shaped lesion’ in the lower lobe of left lung.

**Figure 3.**
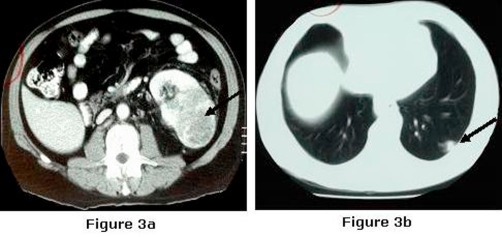
(**A**) Computed tomography of abdomen and thorax. showing left sided renal cell carcinoma, (**B**) Computed tomography of abdomen and thorax Left sub-pleural lesion suspicious of metastasis.

A chest radiograph obtained during his barium swallow identified a “coin shaped” lesion in the periphery of the left lower lobe of the lung (Figure 2). On the same day his ultrasound scan of the abdomen detected a mass arising from the left kidney measuring 8x6x6 cms. A computed tomography scan confirmed a large tumour arising from the left kidney with renal vein involvement, and a left sub-pleural lesion of uncertain nature, but suspicious of a metastasis (Figure 3).

Immunological tests were negative for autoantibodies, extractable nuclear antigens, anti-centromere, anti-topoisomerase-I (Scl-70) as well as the anti-neuronal antibodies (anti-Hu, anti-Yo and anti-Ri).

The patient was given regular metoclopramide and dietary input to optimise his nutritional status. He underwent a left radical nephrectomy for a poorly differentiated renal cell carcinoma (stage T3b, N0, Mx) at the end of December 2003. He was discharged home after an uneventful post-operative course.

He was reviewed by the cardiothoracic surgeons and respiratory physicians with regards to the lung lesion. Serial CT scans showed that the lesion slowly decreased in size and then disappeared altogether over a 5 month period. A PET scan was normal 6 months post-operatively. The lesion was therefore deemed to be benign. Surveillance CT scans are been carried out every 12 months, as pulmonary metastases from renal cell carcinomas can regress spontaneously following removal of the primary lesion, yet the long-term consequence is unknown [2].

The dysphagia that prompted the initial assessment had resolved completely by the time of review in the gastroenterology clinic a few weeks after surgery. Repeat oesophageal manometry and pH study at 12 months after presentation was normal. His renal functions have remained stable. He remains well and annual CT scan surveillance has remained normal to date.

## Discussion

Many types of malignancies are associated with psuedoachalasia leading to dysphagia. They may result in intra-luminal narrowing, extra luminal compression, metabolic disturbance and paraneoplastic effects such as endocrine abnormalities eg: hypercalcaemia. There may also be a paraneoplastic interference with the myenteric neural plexus that controls and co-ordinates the swallowing mechanism. The myenteric plexus may be infiltrated directly, or attacked by autoantibodies generated by the host's immune system in response to tumour surface antigens [3,4]. Although these autoantibodies may suppress tumour development, they can cross react with antigenic proteins on or in neurones leading to nerve injury and damage. These effects can be more disabling than those of the tumour itself. The symptoms produced depend on the particular area of the nervous system attacked, and range from cerebral and cerebellar syndromes, to ophthalmoplegia, and peripheral nerve injury [5]. Achalasia has also been reported due to anti-neuronal antibodies (anti-Hu and to a lesser extent anti-Yo and N-type Ca^2+^ channel anti-neuronal antibodies [6]). Although there are more than ten detectable anti-neuronal antibodies found in the sera of patients with neurological paraneoplastic syndromes, failure to find an antibody does not mean that one does not exist, only that current techniques cannot identify it [5].

It is well recognised that renal cell carcinomas may present in a variety of ways. About five percent of the cases present as paraneoplastic syndromes including anaemia, amyloidosis, hyperviscosity syndromes and neuropathies. Dysphagia in renal cell carcinoma has been reported due to external oesophageal compression [7] and oesophageal metastasis [8,9]. This is the first reported case of dysphagia which is likely to be a consequence of paraneoplastic autonomic neuropathy secondary to a renal cell carcinoma. Furthermore, it resulted in the diagnosis and treatment of an asymptomatic neoplasm. Although the anti-neuronal and anti-Hu antibody has been detected in patients with renal cell cancer and coexisting small cell lung cancer [10], it was not found in this patient. However, the complete resolution of dysphagia symptoms and return to normal physiology following resection of the tumour leads us to believe that the lack of identifiable antibodies is related to their lab detection limitations rather than the absence.

### Limitation

In our patient's case, the mild proteinuria detected on urine dipstick at initial presentation was overlooked. Looking back through literature it seems there may be an association between ‘significant (nephrotic range) proteinuria’ and renal cell cancer. Proteinuria may be a diagnostic sign of renal vein involvement with a tumour thrombus in patients with renal cell cancer [11]. Also pre-operative significant proteinuria may be a risk factor in predicting post-nephrectomy decline in renal function after many years [12,13]. The proposed causes of proteinuria may vary from venous congestion and glomerular architectural destruction to paraneoplastic effects from other tumours (eg. oesophageal, colonic, transitional cell carcinoma etc) causing glomerulonephropathies. In our patient's case, the part of the resected kidney that was not apparently involved by the cancer was macroscopically normal in appearance although histologically it showed changes of focal chronic inflammation with fibrosis, as a result of obstruction. This could explain his mild proteinuria. Therefore it would seem reasonable not to ignore simple or isolated proteinuria in clinical practice.

## Conclusion

Although the anti-neuronal antibodies (anti-Hu, anti-Yo and anti-Ri) were not detectable, we believe that this patient's dysphagia was a paraneoplastic manifestation of the renal cell tumour, an association that has never been previously reported. We suggest that alternative causes for dysphagia must be sought if initial investigations are unhelpful. Importantly, this must include the consideration of a paraneoplastic process secondary to an occult neoplasm.

## Abbreviation

None.
